# Report of Case of Cardiospasm

**Published:** 1911-02

**Authors:** C. A. Dexter

**Affiliations:** Columbus, Ga.


### REPORT OF A CASE OF CARDIOSPASM.

By C. A. Dexter, M. D., Columbus, Ga.

Patient, J. P., mulatto cook, aged about 50 years, says she had always enjoyed good health and had never had attack like the present one. Suffered occasionally with mild attack of ordinary indigestion, promptly relieved by a little soda.

Had been cook in same family about 18 years and they gave same report as to her general health. (Family history negative.)

Present trouble: On day when symptoms began, arose feeling as well as ever, walked a half a mile to the house and cooked breakfast; about two or three hours later began to feel pain in epigastrium and lump in throat; soda gave no relief this time; on consulting me, thinking symptoms were due to indigestion and biliousness, I ordered small hourly doses of calomel.

Next day, to my surprise, she called on me and said that symptoms were not only no better, but decidedly worse, and pain in epigastrium radiated through to between shoulder blades and was much more acute and that swallowing was so painful that she had refrained from eating and had even taken but little water to drink; said that the pain was constantly present, and became much more severe when she tried to swallow and that swallowing liquids was just as painful as solids.

Pain was so severe as to keep her awake all night; gave a hypodermic of morphine, hoping to relieve spasm and permit patient to get rest; next morning said she had got a little rest but pain and discomfort were certainly no better. Attempted to pass stomach tube and had no trouble until what must have been a point near cardia was reached, when tube stopped abruptly and no amount of force or strategy would move it past this point.

Patient had only taken water in small quantities, and had taken practically nothing in the way of food; said pain was too severe, and had no appetite anyhow; next day symptoms were not quite as severe, but the day following all symptoms were more marked; up to this time there had been no vomiting.

I insisted on the patient's taking something in the way of liquid food and went again several hours later and attempted to use a stomach tube of larger size and a little more stiff. Before tube reached the stomach, there was vomiting and some vegetable soup came up, and more after the stomach was reached. I learned that she had just taken this about a half-hour previously, and amounted to over a pint.

After this the pain was more slight, swallowing more comfortable, she began to eat, but it was several days before she felt that everything was all right.

This was three months ago and so far has had no signs of recurrence.

